# Geometric discord: A resource for increments of quantum key generation through twirling

**DOI:** 10.1038/srep13365

**Published:** 2015-08-26

**Authors:** Xiaohua Wu, Tao Zhou

**Affiliations:** 1 College of Physical Science and Technology, Sichuan University, Chengdu 610064, China; 2 School of Physical Science and Technology, Southwest Jiaotong University, Chengdu 610031, China

## Abstract

In the present work, we consider a scenario where an arbitrary two-qubit pure state is applied for the quantum key generation (QKG). Using the twirling procedure to convert the pure state into a Werner state, the error rate of the key can be reduced by a factor of 2/3. This effect indicates that entanglement is not the sufficient resource of QKG protocol since it is not increased in the twirling procedure. Based on the fact that the geometric discord is increased in the twirling procedure, we argue that the geometric discord should be taken as a necessary resource for the QKG task. Besides the pure state, we also give other two types of mixtures where twirling may increase the discord and reduce the error rate of the generated key.

How to quantify and characterize the nature of correlations in a quantum state, has a crucial applicative importance in the field of quantum information processing^1^ beyond the fundamental scientific interest. It is well known that a bipartite quantum state can contain both classical and quantum correlations. Quite recently, quantum discord was introduced as a more general measure of quantum correlation^2^,^3^ beyond the quantum entanglement^4^. Since it was regarded as a resource for quantum cryptography^5^, quantum computation^6^, quantum state merging^7^,^8^, and remote state preparation^9^, quantum discord has attracted much attention in recent works^6^,^7^,^8^,^9^,^10^,^11^,^12^,^13^,^14^,^15^,^16^,^17^,^18^,^19^,^20^,^21^,^22^,^23^,^24^,^25^,^26^,^27^,^28^,^29^,^30^,^31^,^32^,^33^,^34^.

Among all the known quantum tasks, quantum key distribution (QKD) is one of the most important cases that have been widely discussed in both the theoretic and experimental aspects^35^. In 1984, Bennet and Brassard^36^ firstly proposed the QKD protocol. In 1991, Ekert proposed a QKD protocol independent on the BB84 paper^37^, and it is also called the Einstein-Podolsky-Rosen (EPR) protocol since the maximally entangled states, or the EPR pairs, are used to complete the task^35^.

In the present work, we develop a generalized EPR protocol where an arbitrary two-qubit state is applied for the quantum key generation (QKG). The error rate of the generated key can be taken as the figure of merit for this task. In the BB84 protocol, the key is sent from Alice to Bob^36^, but in our work, the key is generated in a different scheme: The keys are undetermined until Alice or Bob performs a measurement on their parts, respectively.

A pure state can be converted into a Werner state in a twirling procedure, and it is interesting that simultaneously the error rate of the key can be reduced by a factor of 2/3 in our QKG scheme. It has already been known that twirling can never increase the entanglement, and therefore, the observed effect, where twirling effectively improves the performance of the pure state in QKG protocol, shows that entanglement is not the sufficient resource for this task. Instead, the geometric discord can be increased in the twirling procedure, and this indicates that the geometric discord may be taken as a necessary quantum resource in the QKG protocol. For the general two-qubit case, we obtain the relation between the error rate and the geometric discord. Based on this, the observed effect may be well explained by the fact that the geometric discord of the pure state can indeed be increased by twirling. Furthermore, we give other two types of mixed states, where twirling may increase the discord and reduce the error rate of the generated key at the same time.

## The EPR protocol for QKD

To process on, we should first notice that an arbitrary two-qubit state *ρ*, which lies in the Hilbert space 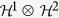 with each 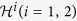 being a two-dimensional space, can always be expressed as


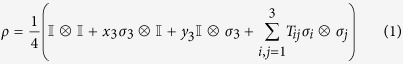


in a fixed basis carefully chosen, where 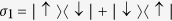, 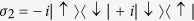, and 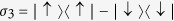 are the Pauli operators, and 
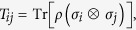
 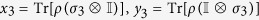. Assume that the states above consist of two spin-1/2 particles labeled by 1 and 2, and Alice measures particle 1 with a fixed observable *σ**a*** = ***σ*** · ***a*** while Bob performs a measurement on the particle 2 with the observable *σ*_***b***_ = ***σ*** · ***b***, where ***a*** and ***b*** are two unit vectors. Then, *a joint measurement for the observable σ*_***a***_ ⊗ *σ*_***b***_ *is called to be optimal if and only if* 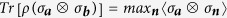. For simplicity, hereafter, we denote 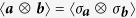. Now four probabilities *ω* _±_ _±_ (***a***, ***b***) can be introduced, *i.e*., *ω* _+_ _+_ (***a***, ***b***) is the corresponding probability in the case that the measurement results for both particles are positive, when the joint measurement *σ*_***a***_ ⊗ *σ*_***b***_ has been performed. Then, for an arbitrary two-bit state *ρ*, one should have


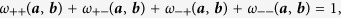


and the correlation function, 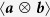, can be expressed as


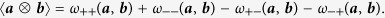


With the optimal measurement defined above, the maximally entangled states are the ones satisfying 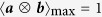 for an arbitrary vector ***a***.

Now, we come to the EPR protocol for QKD. It is well known that maximally entangled states can be applied to generate a randomly distributed key as in the following arguments^38^:

1. A large amount of EPR pairs shared by Alice and Bob are prepared, and Alice (Bob) randomly measures her (his) particle of a EPR pair with *σ*_***a***_ or *σ*_***a′***_ (*σ*_***b***_ or *σ*_***b′***_), where ***a***′⊥ ***a***, ***b***′⊥ ***b***;
2. After sufficient runs of measurements have been performed, Alice and Bob exchange the information about the observable used in each run over a public channel;
3. The experimental data from the measurements for the observables *σ*_***a***_ ⊗ *σ*_***b′***_ and *σ*_***a′***_ ⊗ *σ*_***b***_ are discarded. In other words, the remaining data come from the measurements performed by the observables *σ*_***a***_ ⊗ *σ*_***b***_ and *σ*_***a′***_ ⊗ *σ*_***b′***_;
4. Finally, by arranging their own remaining experiment data in time sequence, each observer can obtain a random key, a long string of symbols like “
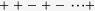
”.

Usually, the maximally entangled state may be chosen to be 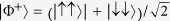. In the present paper, we use the same symbol Φ^+^ o denote the density operator of the pure state, 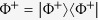, if no confusion is caused. The QKD task realized in the EPR protocol above was proven to be equivalent to the BB84 scheme^38^. Furthermore, one may verify that the two-particle state 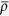,


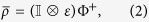


can be also applied to complete the QKD task. For instance, suppose that Alice randomly selects the measurement from *σ*_***x***_ or *σ*_***y***_ (***x*** = (1, 0, 0) and ***y*** = (0, 1, 0)), the EPR protocol realized with 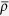 is equivalent to the BB84 scheme where Alice prepares particles in a random sequence of the four states, 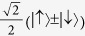 and 
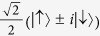
, and sends them to Bob via a noisy quantum channel *ε*.

## The quantum key generation protocol

In the present work, we shall consider a more general scenario where the arbitrary two-qubit state in Eq. (1) is applied for the quantum key generation (QKG). Compared with the EPR protocol, the differences come from the following two aspects:

1. To get a random distributed key, it is necessary that the two eigenvectors of *σ*_***a***_ (*σ*_***a′***_) should appear with equal probability in each measurement, which means, for the state *ρ* in Eq. (1), it is required that ***a*** (***a***′) should be chosen in the *x* − *y* plane of the Bloch sphere. For simplicity, we choose that ***a*** = ***x*** and ***a***′ = ***y***.
2. The keys in Alice’s site may be different from the ones in Bob’s site, and the following two measurable quantities, 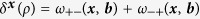, and 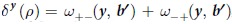, can be used to characterize the discrepancy. The physical meaning of *δ*^***x***^ and *δ*^***y***^ are clear: They are the probabilities that Alice’s measurement result is different from the one of Bob’s when the joint measurement *σ*_***x***_ ⊗ *σ*_***b***_ and *σ*_***y***_ ⊗ *σ*_***b′***_ are performed, respectively.

Based on the condition that Alice (Bob) selects ***x*** and ***y*** (***b*** and ***b***′) with equal probability, it is reasonable to define *the (average) error rate of the key*, to be


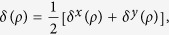


and it can be taken as the figure of merit to quantify the general EPR protocol designed above. With the two equalities, 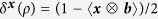 and 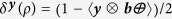, one can obtain


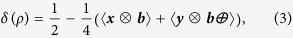


which shows that the error rate is decided by the expectation values of the two observables *σ*_***x***_ ⊗ *σ*_***b***_ and *σ*_***y***_ ⊗ *σ*_***b′***_ introduced before.

Choosing certain local unitary transformations, a bipartite pure state can always be expressed as


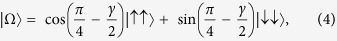


with *γ* a free parameter, 0 ≤ *γ* ≤ *π*/2. When *γ* = *π/*2, 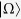 is a product state, 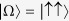. Recently, we have shown that the two-qubit state in Eq. (1) can be expressed in an equivalent form^39^:


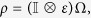


if the reduced density matrix, 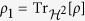, is a mixed state. With the relation above, one may easily find that our QKG protocol cannot be taken as a variation of the known QKD scheme. Under the condition that the quantum channel 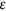 is noiseless, Alice and Bob could get a perfect key via the BB84 or the EPR protocol. However, under the same condition, the key generated by *ρ* always has a non-vanishing error rate, *δ* = sin^2^(*γ*/2), if 0 < *γ* < *π*/2.

## Twirling and its effects

In 1989, Werner gave a one parameter family of twirling invariant states which do not violate the Bell inequality although these states are entangled^40^. Since then, twirling has been widely discussed in many quantum tasks, such as the entanglement distillation^41^,^42^ and quantum process tomography^43^,^44^,^45^. Following the definition in Ref. [42], any two-qubit state *ρ* subjected to the *U* ⊗ U^*^ twirling, where *U* is an arbitrary two-dimensional unitary transformation, can produce a Werner state *ρ*_*W*_(*F*) as


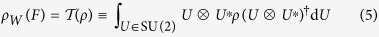


with 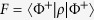. By introducing the four maximally entangled states, 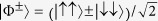 and 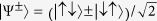, a Werner state *ρ*_*W*_(*F*) in Eq. (5) is


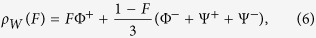


where *F* is a real number, and 0 ≤ *F* ≤ 1. For the two-qubit states, the Werner states are the unique ones which are invariant under the twirling procedure^40^. From the definition in Eq. (5), it is easy to verify that the pure state in Eq. (4) subjected to the twirling can produce a Werner state with *F* = cos^2^(*γ/*2).

In the QKG task developed in the argument above, where an arbitrary two-qubit state is applied to generate a randomly distributed key, there exist some cases that twirling can be used to reduce the error rate of the key. As an important example, by performing twirling on the pure state, the error rate of the key will be effectively reduced,


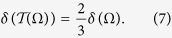


The derivation of this equation is in the following.

First, for the state in Eq. (1), by some algebra, one can obtain


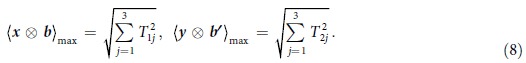


Then, for the pure state in Eq. (4), the density operator can be written as


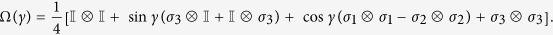


and therefore, with the optimal settings ***b*** = (1, 0, 0) and ***b***′ = (0, −1, 0), we can obtain 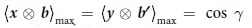. The minimum error rate *δ*(Ω) = sin^2^(*γ*/2).

Meanwhile, the Werner state in Eq. (6) has an equivalent form,


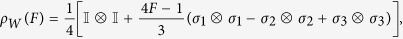


and with the same optimal settings as the pure state, we have 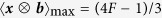. By taking *F* = cos^2^(*γ*/2), the minimum error rate of the pure state after twirling is 
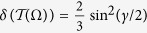
, which exactly gives the result in Eq. (7).

## Geometric discord as a resource for QKG

It has been mentioned before that the effect of twirling shown in Eq. (7) indicates that entanglement is not the sufficient resource to realize the QKG protocol, and hence some other quantum resource beyond entanglement should be responsible for this. In the present work, we argue that quantum geometric discord may be viewed as this kind of quantum resource. Our argument is based on the following two aspects.

(i) For the general two-qubit states, we have the following lemma.

**Lemma 1.** *The geometric discord for a general two-qubit state*, 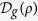
*, is bounded by two optimal values* 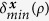 *and* 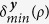 *such that*


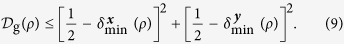


*Proof:* To verify this relation, we should recall the definition of the geometric discord as the first step. If Alice performs an arbitrary projective measurement 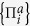 on *ρ*, the final state of the joint system is 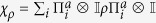. Usually, *χ*_*ρ*_ is regarded as the classic-quantum (CQ) state. With the squared Hilbert-Schmidt norm, ||*A*||^2^ = Tr(*AA*^†^), the geometric discord is defined as 
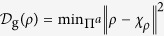
16. Following the result in Ref. [17], this quantity can also be expressed as the difference of two purities,


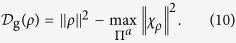


Now, we introduce a special CQ-state 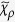,


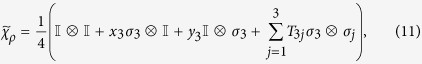


and obviously, this is the final state after that the projective measurement 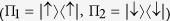 is performed by Alice. With the definition in Eq. (10), one has 
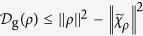
. By jointing it with the Eqs. (3) and (8) and the relation


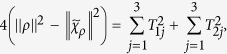


the result in Eq. (9) is easily obtained.

Considering a subset of the two-qubit state where the two correlation functions 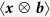 and 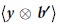 have the same maximum value, say 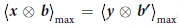, we get a relation between the error rate and the geometric discord,


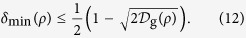


The equality is saturated if 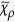 is the closest CQ-state to *ρ*.

As an example, we focus on the so-called 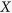-type state,


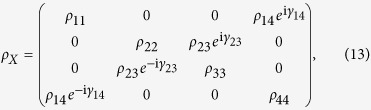


where *ρ*_*ij*_(*i*, *j* = 1, 2, 3, 4) and *γ*_*ij*_ are real positive numbers. The *X*-states constitute a subclass of the general two-qubit state in Eq. (1) with *T*_13_ = *T*_23_ = *T*_31_ = *T*_32_ = 0. Now, the special CQ-state, 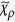, should be


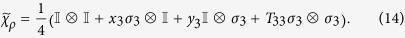


As one of the main results given by Bellomo *et al*.^17^, 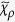 in Eq. (14) should be the closest CQ-state to the state *ρ*_*X*_ in Eq. (13) if 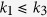, where


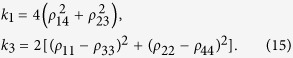


(ii) The effect in Eq. (7) may be well explained by the fact that twirling increases the geometric discord of a pure state. This result is supported by the following two lemmas.

**Lemma 2.** *For a pure state or a Werner state of a bipartite system, the minimal error rate of the key is*


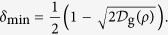


*Proof:* It is easy to see that both the Werner state in Eq. (6) and the pure state in Eq. (4) belong to the so-called 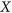-type states. For the pure state, the quantities in Eq. (15) are 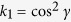 , 
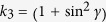
, and 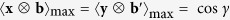, while for the Werner state, 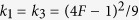, and 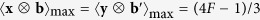. It is obvious that Eq. (12) is saturated for both the pure state and the Werner state, which completes the proof.

**Lemma 3.** *For a pure state in* Eq. (4) *and the Werner state produced by this state subjected to* 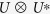 *twirling, the entanglement is the same, while the geometric discord is increased*.

*Proof:* It is well known that twirling is an irreversible operation, and therefore never increases the entanglement of the state^41^. To verify that the entanglement of a pure state is unchanged after a twirling procedure, recall that the entanglement of formation (EoF) is a well-defined measure of the entanglement for a two-qubit state *ρ*^46^


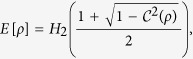


where 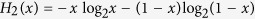 is the binary entropy and 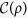 is the concurrence of the state *ρ*. Direct calculation shows that, for the pure state in Eq. (4) and the Werner state in Eq. (6), 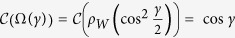. Therefore, 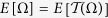, which means the entanglement is the same.

On the other hand, with Bellomo’s result^17^, the geometric discord for *X*-state *ρ*_*X*_ is 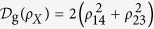 for the case 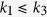. By some simple algebra, one can obtain


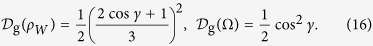


It is clear that the twirling operation on the pure state has increased the geometric discord


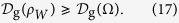


## Non-locality decreased by twirling

In Bell’s celebrited work^47^, it is known that non-locality is a quantum phenomenon which can not be explained by a local-Hidden-variables (LHV) theorem. Usually, one may introduce a Bell operator 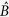 and calculate its expectation 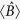 with the LHV theory. With a carefully chosen 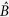, one may find a bound *C*_0_, or equvalently, the Bell inequality 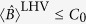 should hold in the LHV theorem. For an example, for the two-qubit states, one may choose 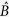 as


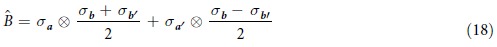


where ***a***, ***a***′, ***b***, and ***b***′ are free unit vectors in the Bloch sphere, and one may get 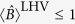, the famous Clauser-Horne-Shimony-Holt (CHSH) inequality^48^.

It has been shown in Werner’s work^40^ that the non-locality is an independent quantum correlation besides the entanglement, and then one may conject that in the QKG task the non-locality and the entanglement can be taken as the sufficient resources. To check this conjecture, we need a strict way to calculate the non-locality for a given two-qubit state, and in this paper, the non-locality of a given state *ρ* is quantified by its ability to violate the CHSH inequality,


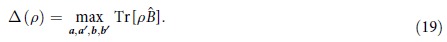


**Lemma 4.** *The non-locality is decreased by twirling*, 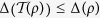.

*Proof:* The twirling procedure in Eq. (5) can be also realized with a set of selected unitary transformations 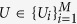
41,


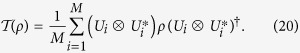


Denote the optimal Bell operator for 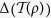 by 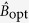, and then


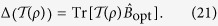


Introduce 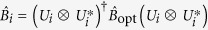, and one can obtain 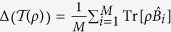 from Eqs. (20) and (21). With the fact that 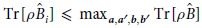 and the definition in Eq. (19), we shall arrive at the desired result.

Based on this, we shall show that the conjecture above does not hold. The reason is quite clear: In the twirling process, both the non-locality and the entanglement is non-increased, and therefore they can not be viewed as the resource of effect in Eq. (7).

## Other cases where twirling reduces the error rate

In the BB84 scheme, Alice prepares particles in a random sequence of the four states, 
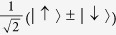
 and 
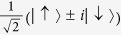
, and sends them to Bob. Suppose Eve, an eavesdropper, use a quantum cloning machine (QCM) to clone these states. The optimal QCM of Eve is described by a unitary transformation 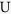 for the jointed system of Bob and Eve^49^,


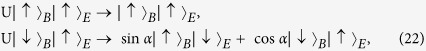


where the free parameter *α* is constrained by 0 ≤ α ≤ π/2. The effect of the optimal QCM on Bob’s state can be represented by an amplitude damping channel *ε*_AD_ with the Kraus operators^50^,


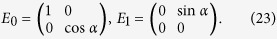


Correspondingly, the scenario discussed above is equivalent to our QKG scheme where a state 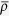,


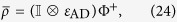


has been applied to generate the randomly distributed keys. For convenience, we rewrite 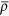 with an explicit form,


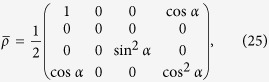


and, obviously, it belongs to the 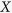-type state defined Eq. (13). With Eq. (15), we get 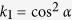 and 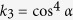. For the present case, 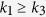, the geometric discord should be


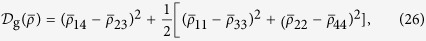


a result given by Bellomo *et al*.^17^. By jointing it with Eq. (24), we shall get 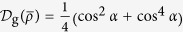.

Meanwhile, with the equivalent form of 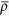,


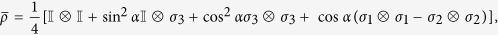


we can obtain 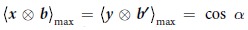 with the optimal settings ***b*** = (1, 0, 0) and ***b***′ = (0, −1, 0). By putting it back into Eq. (3), the minimum error rate of 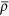 is known to be 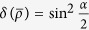.

When 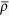 is subjected to twirling, it produce a Werner state, 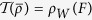, with 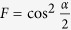. With a simple derivation, one may get 
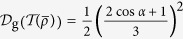
 and 
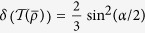
. One can see that, after the state 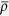 is subjected to twirling, the error rate will be reduced by a factor of 2/3 while its geometric discord is also increased,


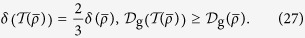


Another example of the two-qubit state, where the error rate of the key can be effectively decreased by twirling, is


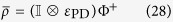


where 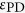 denotes a phase damping channel^1^. The Kraus operators of 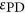 are 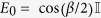 and *E*_1_ = sin(*β*/2)*σ*_3_ (0 ≤ *β* ≤ *π*/2). It can be taken as a model for the situation where a maximally entangled state Φ^+^ is subjected to partial decoherence^2^. With the explicit form


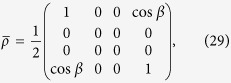


one may check that it belongs to the *X*-type states with 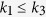. After the twirling has been performed, it produce a Werner state with *F* = cos^2^(*β/*2). With a simple calculation, we get the results: 
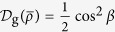
, 
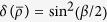
, 
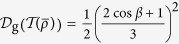
 and 
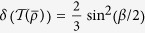
. Obviously, the the relations in Eq. (27) also hold.

In the work of Horodeckis’^42^, it has been proven that the twirling of state 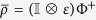, is equivalent to the twirling of the channel *ε*,


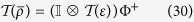


where 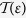 is a depolarizing channel by performing twirling on the initial channel 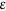. Since the scheme for the twirling of the quantum channel has been developed in recent years^43^,^44^,^45^, one may realize the QKG tasks with 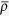 (given in Eq. (24) or Eq. (28)) and 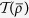, respectively, and the effect of twirling estimated in Eq. (27), 
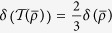
, can be observed in experiments.

## Conclusions and Summaries

In the present work, with the developed QKG scheme, we have demonstrated that twirling can efficiently reduce the error rate of the key generated by some given two-qubit states and argued that the quantum geometric discord may be taken as a necessary resource for the QKG protocol.

From the definition in Eq. (5), we see that twirling is a series of bi-local operations, and it can increase the geometric discord of pure states. It should be noticed that this property of twirling has not been revealed in previous works. Specifically, it has been shown that geometric measure of quantumness of multipartite systems with arbitrary dimension cannot increase under any local quantum channel, if the initial state is pure^51^. However, as it is shown in Eq. (17), the geometric discord is increased when the pure state is subjected to twirling.

Besides the pure states, we also find another two types of mixed states where the twirling can increase the geometric discord and decrease the error rate at the same time. Actually, under which conditions the twirling may increase the geometric discord of the general states is still an open question. Even for the arbitrary two-qubit state, the conditions for applying the twirling procedure to increase the geometric discord and reduce the error rate of the generated key are still unknown. We expect that our results could lead to further theoretical or experimental consequences.

## Additional Information

**How to cite this article**: Wu, X. and Zhou, T. Geometric discord: A resource for increments of quantum key generation through twirling. *Sci. Rep*. **5**, 13365; doi: 10.1038/srep13365 (2015).
